# Characterization of neurocognitive deficits in patients with post-COVID-19 syndrome: persistence, patients’ complaints, and clinical predictors

**DOI:** 10.3389/fpsyg.2023.1233144

**Published:** 2023-10-17

**Authors:** Valeska Kozik, Philipp Reuken, Isabelle Utech, Judith Gramlich, Zoe Stallmach, Nele Demeyere, Florian Rakers, Matthias Schwab, Andreas Stallmach, Kathrin Finke

**Affiliations:** ^1^Department of Neurology, Jena University Hospital, Jena, Germany; ^2^Department of Internal Medicine IV (Gastroenterology, Hepatology and Infectious Diseases), Jena University Hospital, Jena, Germany; ^3^Department of Clinical Neurosciences, University of Oxford, Oxford, United Kingdom; ^4^Center for Sepsis Control and Care (CSCC), Jena University Hospital, Jena, Germany; ^5^Department of Psychology, Ludwig-Maximilians-University, Munich, Germany

**Keywords:** post-COVID, cognitive profile, neuropsychological assessment, infectious diseases, neurocognitive deficits

## Abstract

**Introduction:**

Cognitive symptoms persisting beyond 3 months following COVID-19 present a considerable disease burden. We aimed to establish a domain-specific cognitive profile of post-COVID-19 syndrome (PCS). We examined the deficits’ persistence, relationships with subjective cognitive complaints, and clinical variables, to identify the most relevant cognitive deficits and their predictors.

**Methods:**

This cross-sectional study examined cognitive performance and patient-reported and clinical predictors of cognitive deficits in PCS patients (*n* = 282) and socio-demographically comparable healthy controls (*n* = 52).

**Results:**

On the Oxford Cognitive Screen-Plus, the patient group scored significantly lower in delayed verbal memory, attention, and executive functioning than the healthy group. In each affected domain, 10 to 20% of patients performed more than 1.5 SD below the control mean. Delayed memory was particularly affected, with a small effect of hospitalization and age. Attention scores were predicted by hospitalization and fatigue.

**Discussion:**

Thus, PCS is associated with long-term cognitive dysfunction, particularly in delayed memory, attention, and executive functioning. Memory deficits seem to be of particular relevance to patients’ experience of subjective impairment. Hospitalization, fatigue, and age seem to predict cognitive deficits, while time since infection, depression, and pre-existing conditions do not.

## 1. Introduction

A considerable number of individuals affected by coronavirus disease 2019 (COVID-19), including mild and asymptomatic cases, report long-term cognitive effects, in addition to fatigue and physical symptoms (e.g., Boesl et al., 2021; Lund et al., 2021; Nalbandian et al., 2021; Reuken et al., 2021; Guo et al., 2022; for reviews see Lopez-Leon et al., 2021; Alkodaymi et al., 2022). If symptoms manifest during or after SARS-CoV-2 infection, persist for over 4 weeks without explanation by another diagnosis, the individual is considered to be experiencing post-acute COVID-19 (PACS; Nalbandian et al., 2021). If these symptoms last beyond 12 weeks, they transition into the chronic phase, leading to a diagnosis of post-COVID-19 syndrome (PCS), in accordance with the latest literature and guidelines from the National Institute for Health and Care Excellence (NICE) (Nalbandian et al., 2021; Venkatesan, 2021). A recent study with 355 patients from a post-COVID-19 outpatient clinic^1^ (Stallmach et al., 2022), reported that over 90% reported signs of fatigue and depression and 23% performed below cut-off in a short cognitive screening (Montreal Cognitive Assessment; MoCA). Similar incidences of below cut-off MoCA scores were reported following SARS-CoV-2 infection in a recent population-based study (Hartung et al., 2022).

The pathophysiologic mechanisms behind cognitive dysfunction in PCS are yet to be fully understood, however evidence is accumulating, which points towards direct and indirect effects of long-term tissue damage in all organ systems, including neurologic, cardiac, and pulmonary injury, as well as ongoing inflammatory processes and autoimmune responses (ref. Yong, 2021; Davis et al., 2023). In line with reports of cognitive dysfunction in PCS, longitudinal data from the UK Biobank revealed regional gray matter changes in survivors of COVID-19, as compared to non-infected controls (Douaud et al., 2022).

Long-term cognitive deficits hold significant clinical importance, as they strongly impact on patients’ daily functioning, employment, and the ability to return to work, and thus, constitute a large disease burden (Davis et al., 2021). A characterization of the cognitive profile in PCS and the relationships of deficits in different domains with subjective cognitive complaints and relevant clinical variables is of the essence. This could aid the understanding of the underlying pathogenic mechanisms and improve knowledge of the course of the syndrome. Overall and general cut-offs for short cognitive screens, such as the MoCA are not suitable for such analyses, however.

Initial evidence from studies using more comprehensive test batteries point towards deficits in the domains of attention, memory, and executive functioning following SARS-CoV-2 infection (e.g., Hampshire et al., 2021; Miskowiak et al., 2021; Bungenberg et al., 2022; García-Sánchez et al., 2022; for review see Bertuccelli et al., 2022). However, in these studies, samples were either small or assessed remotely in uncontrolled settings, participants did not meet criteria for the diagnosis of initial infection or PCS (Venkatesan, 2021) consistently, and/or healthy control groups were missing.

For the reliable identification of a domain-specific neuropsychological profile and the clinical factors influencing the cognitive deficits, it is crucial to assess large, well-defined patient groups with appropriate assessment tools. Furthermore, comparisons with socio-demographically equivalent, healthy groups are needed to control for the potential influence of generally increased psychological stress under conditions of a pandemic. However, the use of comprehensive neuropsychological batteries, particularly in a standardized, in-person setting is not easily scalable, as it is time-consuming regarding application, scoring, and interpretation, and requires specialized staff.

The present study used a clinically viable, time- and cost-effective alternative. The Oxford Cognitive Screen-Plus^2^ (OCS-Plus) is a tablet-based screening tool, which bridges the gap between short-from screens and comprehensive neuropsychological batteries, in terms of resource-efficiency and good psychometric properties, as measured by a test–retest protocol as well as convergent and divergent measures comparisons (ref. Demeyere et al., 2021). It facilitates a more detailed screening of domain-specific cognitive functions and the establishment of a profile of affected and spared domains in subclinical and clinical populations (Humphreys et al., 2016; Demeyere et al., 2021). The OCS-Plus has been validated for the detection of subtle cognitive deficits in a healthily aging population, sub-acute stroke, and chronic stroke survivors (Demeyere et al., 2021; Webb et al., 2022). This study is the first to assess cognitive performance in a PCS cohort using the OCS-Plus. Its use requires little training from operating staff and outcome measures are scored automatically.

The first aim of this study was to elucidate the cognitive profile associated with PCS by assessing all potentially relevant cognitive domains, i.e., memory, attention, and executive functions, in a large, clinical sample in comparison to a healthy control group, equivalent in terms of age, sex, and education. The second aim was to establish relationships between affected cognitive domains and subjective cognitive complaints, as well as relevant clinical variables, such as initial disease severity, time since infection, age, depression, fatigue, and comorbidities to identify predictors of specific cognitive deficits in this clinically referred, well-defined PCS cohort.

## 2. Methods

### 2.1. Participants

A total of 282 patients and 52 healthy controls were included in this study. We included all patients who presented to the post-COVID-19 outpatient clinic at Jena University Hospital (Germany) between August 2020 and March 2022 and who had previously been confirmed positive for SARS-CoV-2 using a PCR-test, were willing and able to give informed consent, and were capable of taking part in the assessment. We further only included participants in either group, who did not have a history of relevant neurological or severe psychiatric disorders potentially impairing cognition or relevant vision and hearing problems, and who were between the ages of 18 and 65. We chose the upper age limit to avoid any issues pertaining to age-associated neurodegenerative processes. Of 399 patients, who initially presented to the clinic, met inclusion criteria and consented to their participation, data for 76 patients is not available due to either technical difficulties before or during testing, data for 39 patients is unavailable due to logistical issues or constraints in the clinical setting, and two participants withdrew consent after testing. For a patient-only regression analysis with six predictor variables, we have 80% power to detect effects larger than *R*^2^ = 0.05 with our smallest sub-sample (alpha = 0.05). Based on the fact of relatively low variability and near ceiling performance on the relevant domain scores of the OCS-Plus by healthy, largely elderly participants (see Table 8 in Demeyere et al., 2021), we expect our smaller, but socio-demographically comparable control group to strike the balance between sufficiency to represent healthy variability and resource efficiency.

### 2.2. Assessment

Patients underwent structured anamnesis including medical history, basic socio-demographic data, and subjective cognitive complaints. All participants completed the depression module of the Patient Health Questionnaire (PHQ-9; Kroenke et al., 2001), the Fatigue Assessment Scale (FAS; de Vries et al., 2004) and the Brief Fatigue Inventory (BFI; Mendoza et al., 1999). The PHQ-9 consists of nine items assessing severity of depression symptoms. The FAS measures severity and impact of fatigue with 10 items targeting both physical and mental aspects of fatigue, as well as their impact on daily activities. The BFI consists of nine items, with a focus on the impact of fatigue on daily functioning and mood. In the same session, cognitive functioning was assessed using the Oxford Cognitive Screen-Plus (Demeyere et al., 2021), which consists of nine^3^ subtasks: Picture Naming, Semantics, Orientation, Word Memory Encoding, Delayed Recall, Trails, Episodic Recognition, Figure Copy, and Cancellation (see Demeyere et al., 2021 and Appendix Table 1).

Assessment, which takes approximately 25 min, is completed using a stylus pen on a tablet computer. From the OCS-Plus subtasks, six domain scores may be calculated: Naming and Semantic Understanding (Picture Naming + Semantics), Memory Encoding (Encoding 1 + Encoding 2), Delayed Memory (Delayed Recall + Delayed Recall and Recognition), Praxis (Figure Copy + Figure Recall), Attention (Cancelation + Invisible Cancelation), and Executive Functioning (Trails Executive Score – Cancelation) false positives (Demeyere et al., 2021).

### 2.3. Statistical analysis

We compared socio-demographic variables, fatigue, and depression scores between patients and healthy controls using *t*-tests with Welch correction to account for the difference in sample sizes. Sex ratios were compared using a chi-squared test with Yates’ continuity correction. Raw OCS-Plus scores were standardized using *z*-transform, and, if required, recoded to achieve uniformity across subtasks, with higher values reflecting better performance. Performance on the OCS-Plus subtasks and overall domain scales was compared between controls and patients using Wilcoxon rank sum tests with continuity correction, a method robust against outliers, and suitable to data which violate assumptions of parametric tests. For *t*-tests, Cohen’s *d* was used to quantify effect sizes and calculated as the difference between group means divided by the pooled standard deviation, with *d* ≥ 0.2, *d* ≥ 0.5, and *d* ≥ 0.8 indicating small, medium, and large effects. For Wilcoxon tests, the *r* value was calculated as *Z* divided by the square root of the number of observations, with *r* ≥ 0.1, *r* ≥ 0.3, and *r* ≥ 0.5 indicating small, medium, and large effects. For Wilcoxon *r*, bootstrap confidence intervals are reported (BCa; 1,000 replications). We corrected for multiple comparisons using the Benjamini–Hochberg procedure (FDR; *Q* = 5%), for all group comparisons. Based on the current state of the literature, we selected subtasks and overall domain scores as particularly relevant to our analyses, which capture the domains of attention, memory, and executive functioning. As we expected patients to perform worse in these domains, we used one-tailed tests (alpha = 0.05). We examined how many patients fell below a cut-off of 1.5 standard deviations below the healthy sample means on the domains of interest. The cut-off value was chosen based on standard neuropsychological practice for denoting mild cognitive impairment (Bondi et al., 2014), which also corresponds to values used in previous studies on cognitive impairment in PCS (e.g., Herrera and González-Nosti, 2022; Herrera et al., 2023). To take into account the heterogeneity of symptoms, particularly of cognitive complaints, we split patients into two groups: those who report memory or concentration problems (high complainers) and those who do not complain of these symptoms (low complainers). These groups were compared in terms of their cognitive performance on the domain scales of the OCS-Plus. To explore predictors of attention, memory, and executive functioning problems as part of PCS, we performed multiple linear regression analyses within the patient cohort. We included initial disease severity, age, days since infection, fatigue, and relevant comorbidities as predictors of performance on the OCS-Plus. The need for hospitalization, i.e., outpatient versus inpatient treatment, was used as a proxy for initial disease severity. Comorbidities were included as an index of five binarized pre-existing conditions: hypertension, coronary heart disease, chronic heart failure, diabetes mellitus, and psychiatric disorders (range: 0–5). To estimate generalizability of the models, we computed nonparametric bootstrap (2,000 replications) confidence intervals around the coefficients. The significance threshold for the overall model and coefficients was set to alpha = 5%. To ensure robustness, coefficients were considered significant only when their bootstrap confidence intervals further did not overlap with zero. We subsequently separated patients into two groups for each pre-existing condition, i.e., condition “present” or “not present,” and compared groups on each cognitive domain to assess the effect of the individual conditions. Analysis was performed using R version 4.2.0 (R Core Team, 2022). Packages used for statistical analyses were *stats* (v4.3.1), *effsize (v0.8.1), rcompanion* (v2.4.30), and *boot* (v1.3–28.1).

### 2.4. Ethics

Written, informed consent was obtained from all participants. The study conforms with the World Medical Association Declaration of Helsinki and received approval from the ethics committee of the Jena University Hospital [amendment to 5,082–02/17].

## 3. Results

### 3.1. Socio-demographic and clinical description of healthy controls and post-COVID-19 patients

Socio-demographic information and self-reported fatigue and depression symptoms for both groups are presented in Table 1, clinical patient data are presented in Table 2. There were no differences between groups in terms of age (*t* = −0.79, *p* = 0.43), education (*t* = 1.72, *p* = 0.09), or sex ratios (chi-squared = 0.52*, p* = 0.47). During the anamnestic interview, 69.9% of patients complained of attention and 58.9% of memory problems. 55.7% of patients complained of both attention and memory problems. The two fatigue questionnaires were highly correlated (Pearson’s *r*[329] = 0.78, *p* < 0.001). Given that the FAS measures various aspects of fatigue, including physical, emotional, and cognitive dimensions, and is more broadly applicable than the BFI, only the results from the FAS will be used for further analysis. As compared to controls, patients scored significantly higher on the FAS (controls, *M* = 17.3, *SD* = 4.81; patients, *M* = 31.27, *SD* = 9.05; *d* = −1.63, 95% CI [−1.96, −1.31], *p* < 0.001) and on the PHQ-9 (controls, *M* = 3.92, *SD* = 2.93; patients, *M* = 10.69, *SD* = 5.57; *d* = −1.3, 95% CI [−1.6, −0.97], *p* < 0.001; see Appendix Table 2 for complete results).

**Table 1:** Socio-demographic data and self-reported fatigue and depression symptoms by group.

**Variable**	**Controls**	**Patients**	**Missing** ^**a**^	***p***
No. (%) with data	52 (16)	282 (84)	–	–
Age, mean (SD, range), years	45.62 (10.15, 22–65)	46.84 (11.30, 18–65)	0/0	0.433
Sex, no. (%), female/male	31/21 (60/40)	186/96 (66/34)	0/0	0.47
Education, mean (SD, range), years	15.28 (1.97, 11–18)	14.76 (2.07, 10–18)	0/28	0.09
Fatigue, mean (SD, range), raw score	17.30 (4.81, 10–32)	31.27 (9.05, 11–50)	2/0	<0.001
Depression (SD, range), raw score	3.92 (2.93, 0–11)	10.69 (5.57, 0–29)	2/0	<0.001

^a^Missingness reported as *n*(controls/patients); SD, standard deviation.

**Table 2:** Clinical patient data for acute SARS-CoV-2 infection and pre-existing conditions.

**Variable**	**Distribution**	**Missing**
*All patients*		
Hospitalization, No. (%), outpatient/inpatient	215/67 (76/24)	0
Weeks since infection, M (SD, range)	37.3 (17.6, 12–104)	0
WHO severity grade, No. (%)		1
1	3 (1.1)	
2	214 (76.2)	
3	12 (4.3)	
4	26 (9.3)	
5	20 (7.1)	
6	0 (0)	
7	6 (2.1)	
Comorbidities		
Cardiovascular diseases, No. (%)	103 (36.5)	0
Diabetes mellitus, No. (%)	14 (5)	0
Psychiatric comorbidities, No. (%)	40 (14.2)	0
*Inpatients only*		
Non-ICU hospital stay, mean (SD, range), days	8.6 (5.5, 2–34)	0
ICU admission, No. (%)	24 (36)	2
ICU stay, mean (SD, range), days	10.5 (11.4, 1–46)	5
Oxygen support, No. (%)	48 (72)	0

WHO, World Health Organization; ICU, intensive care unit; cardiovascular diseases include hypertension, coronary heart disease, and chronic heart failure.

### 3.2. Comparison between patients and healthy controls on OCS-Plus subtasks

Patients scored lower than healthy controls on the tasks Delayed Recall accuracy (*r* = 0.12, 95% CI [0.02, 0.22], *p* = 0.01), Figure Copy accuracy (*r* = 0.1, 95% CI [−0.02, 0.2], *p* = 0.04), Cancelation false positives (*r* = 0.1, 95% CI [0.001, 0.16], *p* = 0.03), and Invisible Cancelation accuracy (*r* = 0.12, 95% CI [0.02, 0.21], *p* = 0.02). However, none of these comparisons survived correction for multiple comparisons. Patients scored numerically lower on the Encoding 2 task (controls, *M* = 0.23, patients, *M* = −0.04), however, there was no variance within the control group on this task. Please refer to Table 3 for full results.

**Table 3:** Performance on the OCS-Plus subtasks per group, descriptive and statistical comparison data.

	**Controls**			**Patients**			**Wilcoxon rank sum test**		
OCS-Plus subtask	***n***	**Mean (SD)**	**Median (Q1;Q3)**	***n***	**Mean (SD)**	**Median (Q1;Q3)**	**W**	***p***	***r* (LL;UL)**
Picture Naming accuracy	52	0.05 (0.79)	0.21 (0.21;0.21)	280	−0.01 (1.04)	0.21 (0.21;0.21)	7,340	0.397	
Semantics accuracy	51	0.09 (0.85)	0.34 (0.34;0.34)	282	−0.02 (1.03)	0.34 (0.34;0.34)	7419.5	0.249	
Orientation accuracy	52	−0.23 (1.39)	0.22 (0.22;0.22)	282	0.04 (0.91)	0.22 (0.22;0.22)	6,913	0.962	
*Encoding 1*	52	0.02 (0.97)	0.72 (−0.8;0.72)	274	0 (1.01)	0.72 (−0.8;0.72)	7158.5	0.475	
*Encoding 2*	52	0.23 (0)	0.23 (0.23;0.23)	279	−0.04 (1.08)	0.23 (0.23;0.23)	-	-	
*Delayed Recall accuracy*	50	0.32 (0.78)	0.47 (−0.22;1.16)	279	−0.06 (1.02)	−0.22 (−0.92;1.16)	8,327	0.013	0.12^a^ (0.02;0.22)
*Delayed Recall and Recognition*	51	0.23 (0.62)	0.45 (0.45;0.45)	279	−0.04 (1.05)	0.45 (0.45;0.45)	7,818	0.052	
*Episodic Recognition accuracy*	51	0.18 (0.82)	0.79 (−0.63;0.79)	280	−0.03 (1.03)	0.79 (−0.63;0.79)	7,768	0.13	
*Trails Executive Score*	51	0.24 (0.7)	0.73 (−0.19;0.73)	278	−0.04 (1.04)	0.42 (−0.5;0.73)	7,918	0.078	
*Processing Speed*	51	−0.19 (1.02)	0.09 (−0.75;0.56)	278	0.04 (0.99)	0.27 (−0.29;0.7)	6,084	0.946	
Figure Copy accuracy	43	0.18 (1.01)	0.54 (−0.34;0.89)	279	−0.03 (1)	0.19 (−0.51;0.54)	7002.5	0.037	0.1^a^ (−0.02;0.2)
Figure Recall accuracy	42	0.06 (0.95)	0.11 (−0.55;0.76)	279	−0.01 (1.01)	0.17 (−0.55;0.76)	6,022	0.386	
*Cancelation accuracy*	51	0.06 (0.89)	0.45 (0.45;0.45)	275	−0.01 (1.02)	0.45 (0.45;0.45)	7,162	0.363	
*Cancelation false positives*	51	0.24 (0.48)	0.34 (0.34;0.34)	276	−0.04 (1.06)	0.34 (0.34;0.34)	7,686	0.03	0.1^a^ (0.001;0.16)
*Invisible Cancelation accuracy*	51	0.32 (0.65)	0.31 (−0.36;0.98)	275	−0.06 (1.04)	0.31 (−0.36;0.98)	8,267	0.018	0.12^a^ (0.02;0.21)
*Invisible Cancelation correct revisits*	52	0.16 (0.75)	0.48 (−0.34;0.75)	276	−0.03 (1.04)	0.2 (−0.34;0.75)	7,782	0.155	

OCS-Plus, Oxford Cognitive Screen-Plus; SD, standard deviation; Q1, 25th percentile; Q3, 75th percentile; SEM, standard error of the mean; *r*, Wilcoxon *r*, effect size; LL, lower limit; UL, upper limit of 95% bootstrap confidence interval; ^a^significant before FDR-correction, ^b^significant after FDR-correction; italicized: subtasks of interest.

### 3.3. Comparison between patients and healthy controls On OCS-Plus domain scales

**Figure 1:**
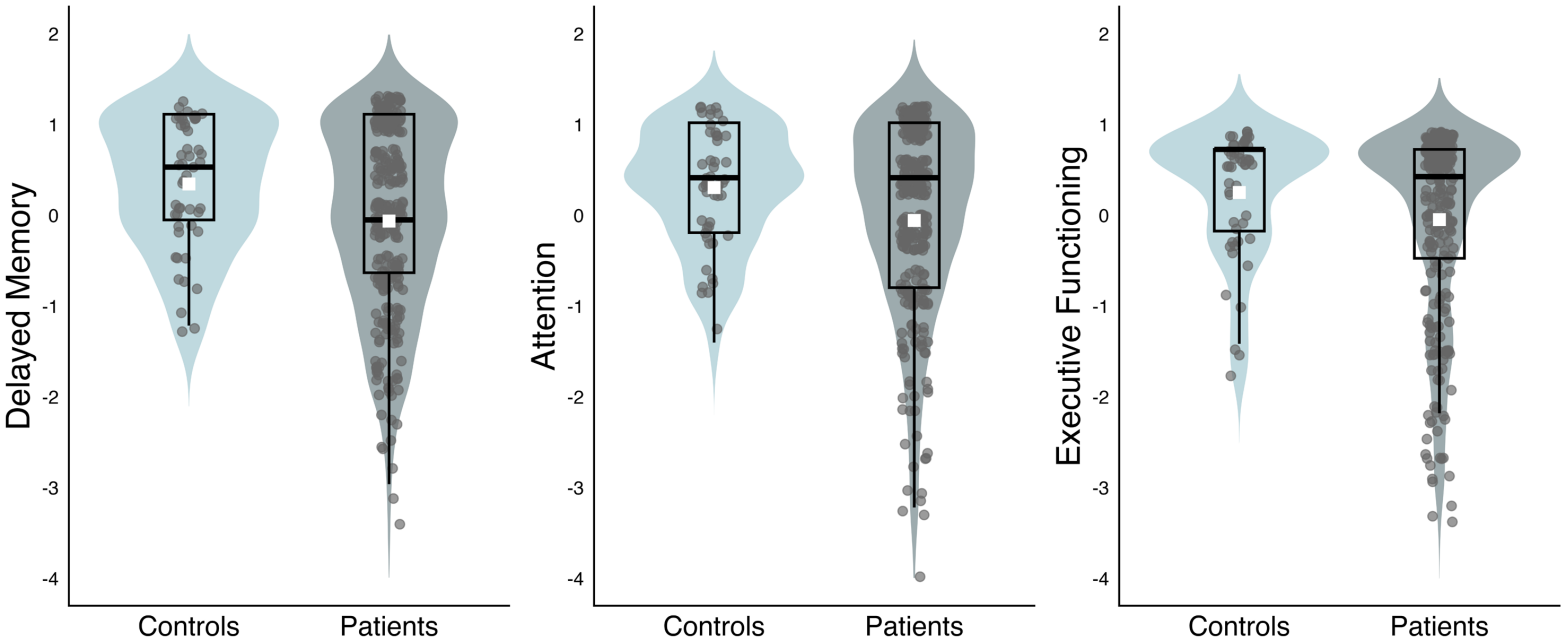
Performance on the OCS-Plus domain scales by controls and patients. Distribution of scores on the OCS-Plus domain scales of Delayed Memory, Attention, and Executive Functioning, per group. Displayed are boxplots, with lines representing the 25th, 50th, 75th percentiles. White squares represent group mean, and gray dots represent individual participants.

Patients scored significantly lower than healthy controls on the scales of Delayed Memory (*r* = 0.13, 95% CI [0.04, 0.23], *p* = 0.008), Executive Functioning (*r* = 0.1, 95% CI [0.002, 0.19], *p* = 0.03), and Attention (*r* = 0.11, 95% CI [0.01, 0.2], *p* = 0.03). All observed effect sizes may be classified as small effects (Cohen, 1988; Gignac and Szodorai, 2016). See Table 4 and Figure 1 for results stratified by group.

**Table 4:** Performance on the OCS-Plus domain scales per group, descriptive and statistical comparison data.

	**Controls**			**Patients**			**Wilcoxon rank sum test**		
OCS-Plus domain scale	***n***	**Mean (SD)**	**Median (Q1;Q3)**	***n***	**Mean (SD)**	**Median (Q1;Q3)**	***W***	***p***	***r* (LL;UL)**
Naming and Semantic Understanding	51	0.1 (0.89)	0.37 (0.37;0.37)	280	−0.02 (1.02)	0.37 (0.37;0.37)	7,478	0.185	
*Memory Encoding*	52	0.09 (0.88)	0.72 (−0.64;0.72)	271	−0.02 (1.02)	0.72 (−0.64;0.72)	7,296	0.322	
*Delayed Memory*	50	0.35 (0.72)	0.53 (−0.05;1.11)	279	−0.06 (1.03)	−0.05 (−0.63;1.11)	8438.5	0.008	0.13^b^ (0.04;0.23)
Praxis	43	0.09 (0.94)	0.21 (−0.58;0.68)	279	−0.01 (1.01)	0.21 (−0.53;0.73)	6,189	0.369	
*Executive Functioning*	50	0.25 (0.69)	0.73 (−0.17;0.73)	275	−0.05 (1.04)	0.43 (−0.47;0.73)	7,945	0.033	0.1^b^ (0.002;0.19)
*Attention*	50	0.31 (0.64)	0.42 (−0.19;1.02)	275	−0.06 (1.04)	0.42 (−0.8;1.02)	8,021	0.027	0.11^b^ (0.01;0.2)

OCS-Plus, Oxford Cognitive Screen-Plus; SD, standard deviation; Q1, 25th percentile; Q3, 75th percentile; SEM, standard error of the mean; *r*, Wilcoxon *r*, effect size; LL, lower limit; UL, upper limit of 95% bootstrap confidence interval; ^a^significant before FDR-correction; ^b^significant after FDR-correction; italicized: domain scales of interest.

### 3.4. Proportional impairment per group on OCS-Plus domain scales

**Figure 2:**
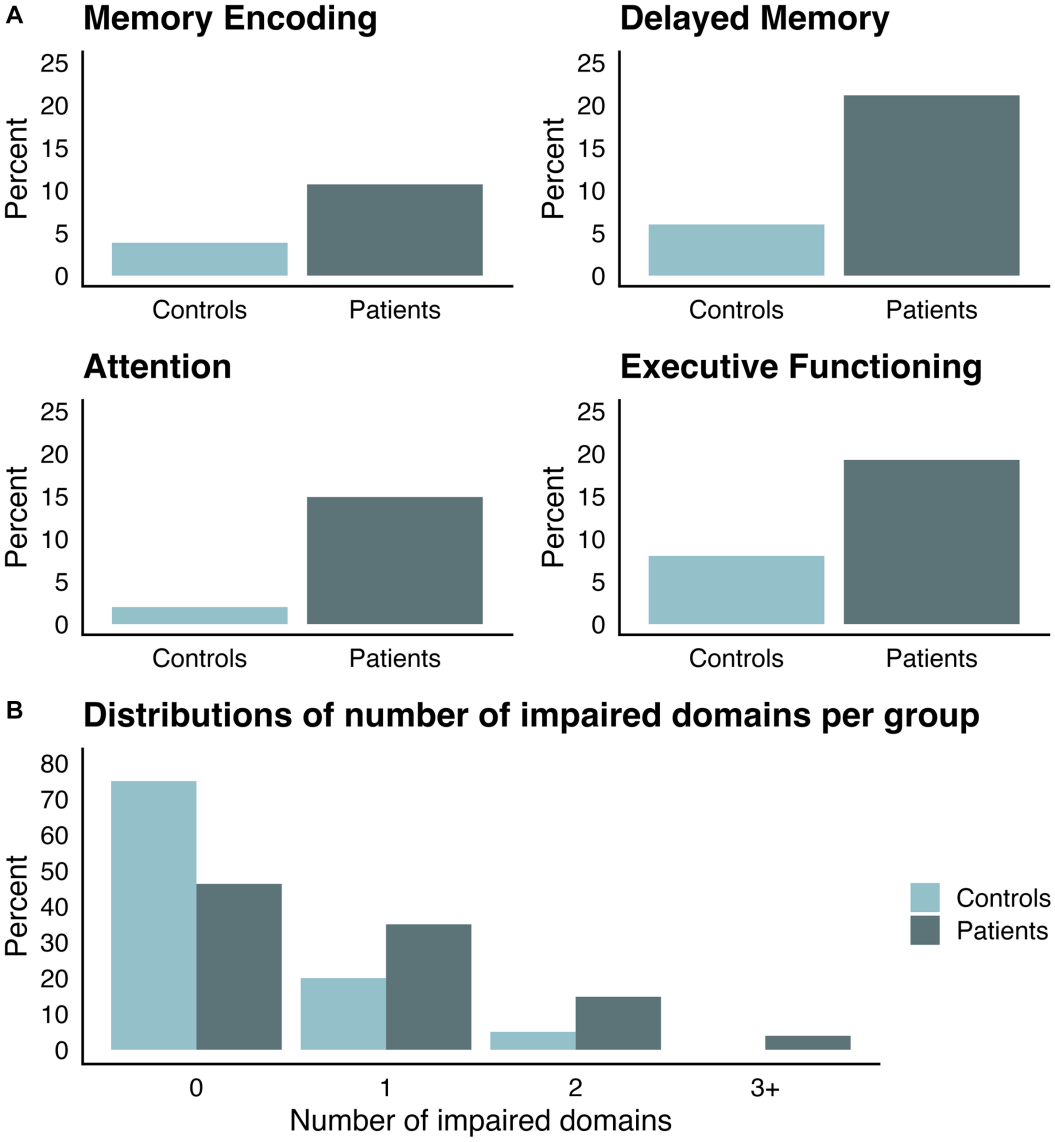
Distributions of controls and patients scoring below cut-off on OCS-Plus domain scores. **(A)** Percentage of participants under the cut-off (1.5 standard deviations below control mean) per group in the domain scores of interest. **(B)** Percentage of participants per group, who fall under the cut-off in no, one, two, or at least three domain scores.

10.7% of patients scored below the cut-off on Memory Encoding (versus 3.85% of controls), 21.15% of patients scored below the cut-off on Delayed Memory (versus 6% of controls), 19.27% of patients scored below the cut-off on Executive Functioning (versus 8% of controls), and 14.91% of patients scored below the cut-off on Attention (versus 2% of controls; see Figure 2A). Out of those patients for whom there is complete data for all domain scores, 53.7% of patients were impaired on at least one domain score (versus 25% of controls), 18.68% scored below the cut-off on at least 2 domains (versus 5% of controls), and 3.89% scored below the cut-off on at least 3 domains (versus 0% of controls; see Figure 2B).

### 3.5. Relationship between subjective cognitive complaints and delayed memory performance

Seventy-six patients reported no cognitive symptoms, 49 reported either attention or memory difficulties, and 157 patients reported both. Performance in the Delayed Memory domain differed between those with and those without subjective cognitive complaints (*W* = 8,669, *p* = 0.024). There were no performance differences on any other domain scale (see Appendix Table 3 for complete results).

### 3.6. Relationships between clinical variables and performance on the domains of delayed memory, attention, and executive functioning

The overall model to predict Delayed Memory performance, with hospitalization, age, days since infection, fatigue, and comorbidities as predictors was significant [*F*(6, 272) = 4.84, *p* < 0.001, *R*^2^adj. = 0.08], hospitalization (*β* = −0.72, 95% CI [−1.21, −0.27]; *p* = 0.006) and age (*β* = −0.03, 95% CI [−0.04, −0.01]; *p* = 0.01) significantly predicted Delayed Memory performance. The model to predict performance on the Attention domain score was also significant [*F*(6, 268) = 4.07, *p* < 0.001, *R*^2^adj. = 0.06], with hospitalization (*β* = −0.78, 95% CI [−1.4, −0.24]; *p* = 0.003) and fatigue (*β* = −0.04, 95% CI [−0.08, −0.01]; *p* = 0.01) as significant predictors. The model to predict performance in the Executive Functioning domain was not significant. Please see Table 5 for full results.

**Table 5:** Coefficient-level estimates for models fitted to estimate variation in (1) Delayed Memory, (2) Attention, and (3) Executive Functioning performance.

	**(1) Delayed Memory**		**(2) Attention**		**(3) Executive Functioning**	
	*Estimate (95% CI)*	*p*	*Estimate (95% CI)*	*p*	*Estimate (95% CI)*	*p*
Intercept	9.564 (8.383, 10.789)	<0.001	59.748 (58.576, 60.844)	<0.001	81.631 (59.821, 99.055)	<0.001
Hospitalization (inpatient)	**−0.724** **(−1.205, −0.265)**	**0.006**	**−0.779** **(−1.402, −0.239)**	**0.003**	**−**2.314 (**−**10.856, 5.074)	0.545
Age	**−0.025** **(−0.042, −0.005)**	**0.013**	**−**0.014 (**−**0.032, 0.003)	0.163	0.069 (**−**0.2, 0.394)	0.642
Days since infection	0.002 (0, 0.003)	0.041	0.001 (0, 0.003)	0.160	**−**0.025 (**−**0.049,**−**0.004)	0.040
Fatigue	**−**0.018 (**−**0.052, 0.013)	0.289	**−0.043** **(−0.075, −0.012)**	**0.011**	0.184 (**−**0.357, 0.676)	0.465
Depression	**−**0.009 (**−**0.06, 0.042)	0.729	0.035 (**−**0.011, 0.086)	0.184	**−**0.029 (**−**0.722, 0.767)	0.943
Comorbidities	**−**0.031 (**−**0.321, 0.239)	0.826	**−**0.055 (**−**0.340, 0.232)	0.691	**−**2.505 (**−**6.728, 1.762)	0.222
Observations	279		275		275	
*R* ^2^	0.096		0.083		0.027	
Adjusted *R*^2^	0.076		0.063		0.005	
Residual Standard Error	1.702		1.668		24.727	
*F* statistic (df)	4.838 (6; 272)	<0.001	4.067 (6; 268)	<0.001	1.223 (6; 268)	0.29

Coefficients and 95% confidence intervals (non-parametric bootstrap, 2,000 replications), bolded: significant estimates, with confidence intervals not overlapping zero; df, degrees of freedom.

On the level of individual comorbidities, those with hypertension performed worse in the Delayed Memory domain. No other comparisons between groups with and without individual comorbidities survived correction. Regression analysis revealed no effect of hypertension on Delayed Memory performance, when controlling for our set of covariates (see Appendix Tables 4–6).

## 4. Discussion

In this study, subtle, yet meaningful deficits in attention, delayed memory, and executive functioning as well as preserved basic orientation, language, and visuo-spatial functions were identified in patients with post-COVID-19 syndrome (PCS). High levels of patients’ subjective cognitive complaints were associated with poorer performance on the OCS-Plus Delayed Memory scale, but not other cognitive domains. In regression analyses, we found significant clinical predictors of memory and attention performance, but none for executive functioning. Specifically, we found that initial disease severity predicted performance in the domains of attention and delayed memory recall, i.e., hospitalized patients performed significantly worse than non-hospitalized patients. Further, older age predicted poorer performance in the delayed memory domain and higher levels of fatigue predicted worse performance in the domain of attention. We found no associations between delayed memory or attention performance and time since infection, depression, or comorbidities.

The identified neuropsychological profile of patients with PCS fits with results of early studies (e.g., Hampshire et al., 2021; Bungenberg et al., 2022; García-Sánchez et al., 2022). However, the present study goes beyond these studies by documenting persisting deficits in a large patient sample with previous SARS-CoV-2 infection confirmed by laboratory testing and fulfilling the NICE criterion of symptom persistence beyond 12 weeks post-infection (Venkatesan, 2021) in comparison to a socio-demographically comparable control group. Moreover, our participants were assessed in a face-to-face setting, i.e., under more controllable, standardized conditions than the remote testing used in a large, population-based study (e.g., Hampshire et al., 2021).

In each of the affected domains — delayed memory, attention, and executive functioning — between 10 and 20% of patients fell below a cut-off of 1.5 standard deviations based on the healthy group distribution. In fact, a substantial number of patients showed domain-level deficits, as more than half of patients scored below the cut-off in at least one major domain score and just under a fifth of patients were impaired on multiple domains. Deficits were found most commonly in the delayed verbal memory domain. This is in line with the finding of predominant left-sided parahippocampal gyrus atrophy in individuals affected by SARS-CoV-2 (Squire and Zola-Morgan, 1991; Douaud et al., 2022). Recent research has started to bring more insight into the structural and functional brain alterations associated with long-term complications of SARS-CoV-2, comprehensively summarized in two recent reviews (Okrzeja et al., 2023; Zhao et al., 2023). The aforementioned longitudinal analysis of structural MRI data from the UK Biobank has revealed tissue changes in orbitofrontal and parahippocampal regions, along with overall brain volume reduction after infection (Douaud et al., 2022). Moreover, alterations in the microstructure of long-reaching white matter tracts have been observed, a finding which is consistent with impaired functioning in tasks requiring network-level communication, such as those assessing executive functioning, attention, and memory (Zhao et al., 2023). Functional alterations, likely of a transient nature, have been observed in frontal, temporal, and parietal regions (Okrzeja et al., 2023; Zhao et al., 2023) These findings align with our results and established links of regional involvement in frontoparietal networks facilitating attentional and executive functioning. Additionally to the described link between (para-)hippocampal gyrus and memory function, it is important to note, that delayed memory recall tasks significantly rely on executive-attentional networks (for review of cognitive networks see Uddin et al., 2019), which appear to have some vulnerability in terms of chronic dysfunction in PCS. Interestingly, patients who reported high levels of subjective cognitive complaints exhibited worse performance in the delayed memory domain only. As we found no associations between other domains and subjective cognitive complaints, memory deficits may play a unique role in how patients perceive their own cognitive functioning. This highlights the need for targeted cognitive rehabilitation interventions to address patients’ subjective experience of daily life impairment. We further found relatively high incidences of deficits in attention and executive functioning, which are among the most commonly reported findings in PCS (e.g., Hampshire et al., 2021; Bungenberg et al., 2022; García-Sánchez et al., 2022).

We further tested for the influence of relevant clinical variables, i.e., the need for hospitalization during acute infection, time since infection, relevant comorbidities, and age. Additionally, we tested for the influence of current symptoms of fatigue and depression, which, in line with previous studies (e.g., Bungenberg et al., 2022; for review see Ceban et al., 2022) were heightened in patients compared to healthy controls. The analyses revealed, firstly, a — relatively small — negative influence of hospitalization on memory and attention performance. While reports regarding the effect of disease severity on cognitive functioning in heterogeneous samples of participants following SARS-CoV-2 infection are inconsistent (e.g., Hampshire et al., 2021; Bungenberg et al., 2022; García-Sánchez et al., 2022), this finding contributes to the understanding of this association with memory and attention in PCS and underscores the significance of incorporating cognitive rehabilitation into broader clinical recovery strategies. Secondly, and in accordance with the well-established decline in verbal memory performance with increasing age (Bopp and Verhaeghen, 2005), our regression analyses revealed a small influence of age on delayed verbal memory performance. In this clinical cohort, both greater disease severity and older age were found to be linked to cognitive dysfunction, as they were found to have independent, small associations with poorer cognitive performance. However, as age is associated with an increased vulnerability to more severe acute infection, it is likely, that their relationships with cognitive dysfunction in PCS are partially inter-related (Cristillo et al., 2022; Crivelli et al., 2022).

Thirdly, fatigue was a predictor of attention performance, which appears to fit within the context of reduced levels of overall brain arousal and cognitive performance, particularly in the domain of attention (Sturm and Willmes, 2001; Boksem et al., 2005; for review see Sara and Bouret, 2012). It should be noted, however, that due to the nature of this study’s design, fatigue was found to partially explain performance variation, without necessarily implying a causal relationship between the factors. As our analyses revealed no associations between cognitive performance and time since infection, they suggest that cognitive deficits in the PCS stage may persist over the long term. However, follow-up assessments are needed to provide more conclusive data regarding the course of domain-specific cognitive functioning. Furthermore, as we did not find evidence for an influence by depressive symptoms or comorbidities, cognitive dysfunctions seem to be due to the infection itself rather than an increased psychological or general health burden.

This study has certain strengths and limitations. Strengths include a large, well-defined PCS patient cohort, a socio-demographically comparable control group, and the use of an innovative, clinically useful tablet-based assessment tool, which combines resource-efficiency and good psychometric properties (Demeyere et al., 2021). While we did not have access to cognitive performance prior to infection, we mitigated this limitation by including a control group, equivalent in terms of age and education, as well as by excluding patients with known relevant neurological or psychiatric disorders. It may further be possible, that recovery, including cognitive functioning, is affected by the specific clinical interventions during the acute infection, such as pharmacological treatment or oxygen supplementation. A recent study has found specific associations between acute symptoms, such as sleep disturbances and headache and cognitive dysfunction at 1 year post-infection (Cavaco et al., 2023). These questions were outside of the current study’s scope, however, studies examining these potential relationships may be illuminating. Our study was prone to selection bias, as only patients with severe enough symptoms to report to a specialized clinic were included. However, this study thus provides a valuable insight into the clinical cohort, for which the health care system needs to be prepared, as numbers of COVID-19 survivors, who continue to experience long-term symptoms, are rising. Notably, our patient sample includes approximately two-thirds women and one-third men, which is consistent with a higher risk of developing PCS in general, being associated with the female sex (Hanson et al., 2022; Tsampasian et al., 2023). While we did not conduct any sex-based analysis in this cross-sectional study, a further investigation on the cognitive trajectory of this cohort should examine the potential role of sex in the recovery of cognitive performance.

This study identified subtle long-term deficits in attention, memory, and executive functioning persisting for more than 3 months in patients with PCS. Memory deficits in particular seem to be associated with subjective levels of impairment. Given the relevance of cognitive deficits for successful reintegration into work and family life, for clinical practice, this indicates a pressing need for the numerous patients suffering from PCS to undergo comprehensive cognitive screening. The OCS-Plus provides a reliable, time- and cost-efficient domain-specific screening. An initial assessment like this can enable clinicians to decide about further diagnostic and treatment steps, such as the necessity to undergo more in-depth neuropsychological assessment in specialized centers, and to start treatment with cognitive rehabilitation interventions, such as occupational therapy or cognitive training targeting affected domains. From a research perspective, our cross-sectional approach should be complemented by a longitudinal study examining the time course of cognitive deficits in the long term and the potential of recovery.

## Data availability statement

The raw data supporting the conclusions of this article will be made available by the authors, without undue reservation.

## Ethics statement

The studies involving humans were approved by Ethics Committee of the Jena University Hospital (Ethikkommission der Friedrich-Schiller Universität Jena an der Medizinischen Fakultät). The studies were conducted in accordance with the local legislation and institutional requirements. The participants provided their written informed consent to participate in this study.

## Author contributions

VK: writing – original draft preparation, data curation, formal analysis, visualization, and data collection. PR: conceptualization and implementation and writing – critical review. IU, JG, and ZS: data collection. ND: methodology. FR and MS: writing – review. AS: conceptualization, supervision, resources, and writing – review. KF: conceptualization, supervision, resources, and writing – review and editing. All authors contributed to the article and approved the submitted version.

## Footnotes

^1^The present study’s patient sample was recruited from the same outpatient clinic, but using different inclusion criteria (see Methods).

^2^While the original OCS is a stroke-specific paper-and-pencil bedside screening test, the OCS-Plus is a computerized elaboration of this tool, with broader clinical and subclinical application.

^3^Due to time constraints in the clinical setting, the OCS-Plus subtask “rule finding” was skipped. Accordingly, scoring for executive functioning differs from the method proposed by Demeyere et al. (2021).
