# Supplementary material for: Characterization of neurocognitive deficits in patients with post-COVID-19 syndrome: persistence, patients’ complaints, and clinical predictors
Source: Front Psychol. 2023 Oct 17;14:1233144. doi: 10.3389/fpsyg.2023.1233144 (PMC10616256; doi:10.3389/fpsyg.2023.1233144)
Supplement: Supplementary file 1 [file Data_Sheet_1.docx]

**Characterization of neurocognitive deficits in patients with post-COVID syndrome: persistence, patients' complaints and clinical predictors**

Kozik, V., Reuken, P., Utech, I., Gramlich, J., Stallmach, Z., Demeyere, N., Rakers, F., Schwab, M., Stallmach, A., Finke, K.

**Appendix**

Table 1: OCS-Plus tasks descriptions

| **Task** | **Description** |  | **Score range** |
| --- | --- | --- | --- |
| Picture Naming | Participants name four images of low-frequency objects. |  | 0 – 4 |
| Semantics | Out of an array of four images, participants identify the correct object based on semantic category. |  | 0 – 4 |
| Orientation | Orientation in time and space is assed using four questions pertaining to the current date, location, and the current German chancellor. |  | 0 – 4 |
| Word Memory Encoding | Participants are tasked with remembering a list of five words, which is presented twice. After each presentation, participants are asked to recall the words (Encoding 1 and Encoding 2). |  | 0 – 5, for Encoding 1 and Encoding 2, each |
| Trails | In two baseline conditions, participants connect circles and squares in increasing and decreasing order of size, respectively. From this, the Processing Speed score is calculated as baseline time divided by baseline accuracy. |  | ∞ |
|  | The switching condition entails connecting circles and squares in a complex, alternating rule, with circles going up and squares going down in size. The Trails Executive Score is calculated as accuracy in the switching condition divided by accuracy in the baseline condition. |  | 0 – 100 |
| Delayed Recall | After the Trails task, participants are asked to recall the encoded words (Delayed Recall). |  | 0 – 5 |
|  | Words, which were not correctly recalled, are presented as part of a multiple-choice array (Delayed Recall and Recognition). |  | 0 – 5 |
| Episodic Recognition | Participants select stimuli (objects or words), which were part of previous tasks out of a multiple-choice array. |  | 0 – 4 |
| Figure Copy | Participants copy a complex figure consisting of 20 geometric elements, each being scored for presence, position, and accuracy. |  | 0 – 60 |
|  | Participants are briefly presented with the same figure again, which then has to be drawn from memory. |  | 0 – 60 |
| Cancellation | Participants are presented with a search array of 60 pictograms of fruit (targets) and vegetables (distractors). Each of the 30 targets needs to be selected once and in the visible condition, selected elements are visibly marked. |  | 0 – 30 (accuracy) and 0 – ∞ (false positive selections) |
|  | In the invisible condition, the pictograms are re-arranged and presented again. Again, each target needs to be selected once, but in the invisible condition, the markings disappear immediately after selection. |  | 0 – 30 (accuracy) and 0 – ∞ (correct revisits) |

Table 2: Questionnaire data for controls and patients

|  |  | **Controls** | | | |  | **Patients** | | | |  | **Wilcoxon rank sum test** | |
| --- | --- | --- | --- | --- | --- | --- | --- | --- | --- | --- | --- | --- | --- |
| **Questionnaire** |  | **n** | **Mean** | **SD** | **SEM** |  | **n** | **Mean** | **SD** | **SEM** |  | ***W*** | ***r*** |
| FAS |  | 50 | 17.30 | 4.81 | 0.68 |  | 282 | 31.27 | 9.05 | 0.54 |  | 1360 | 0.50*** |
| PHQ-9 |  | 50 | 3.92 | 2.93 | 0.41 |  | 282 | 10.69 | 5.57 | 0.33 |  | 2004.5 | 0.44*** |

*Note*. FAS = Fatigue Assessment Scale; PHQ-9 = Patient Health Questionnaire, depression module; SD = standard deviation; SEM = standard error of the mean; *r* = Wilcoxon *r* effect size; ^a^ * p<.5; **p<.01; ***p<.001

Table 3: Performance on the OCS-Plus domain scales in patients with low versus high complaints of cognitive symptoms

|  | **Low complaints** | | | | | **High complaints** | | | | | **Wilcoxon rank sum test** | |  |
| --- | --- | --- | --- | --- | --- | --- | --- | --- | --- | --- | --- | --- | --- |
| OCS-Plus task | n | Mean | Med. | SD | SEM | n | Mean | Med. | SD | SEM | *W* | *p* | *r* |
| Naming and Semantic Understanding | 76 | 0.07 | 0.37 | 0.88 | 0.10 | 204 | -0.05 | 0.37 | 1.07 | 0.07 | 8052.5 | .208 |  |
| Memory Encoding | 73 | 0.05 | 0.72 | 1.10 | 0.13 | 198 | -0.04 | 0.72 | 1.00 | 0.07 | 7764 | .143 |  |
| Delayed Memory | 73 | 0.15 | 0.53 | 0.95 | 0.11 | 206 | -0.14 | -0.05 | 1.05 | 0.07 | 8669 | .024 | 0.12 |
| Praxis | 75 | -0.05 | 0.21 | 1.30 | 0.15 | 204 | 0.00 | 0.21 | 0.89 | 0.06 | 8117 | .217 |  |
| Executive Functioning | 73 | -0.23 | 0.38 | 1.19 | 0.14 | 202 | 0.02 | 0.56 | 0.98 | 0.07 | 6516 | .938 |  |
| Attention | 74 | 0.07 | 0.42 | 0.97 | 0.11 | 201 | -0.10 | -0.19 | 1.07 | 0.08 | 8113 | .118 |  |

*Note*. Med. = Median; SD = standard deviation; SEM = standard error of the mean; *r* = Wilcoxon *r* effect size

Table 4: Group-level descriptive statistics for delayed memory, attention, and executive functioning, stratified by presence of comorbidity, within patient group

|  | Delayed Memory | | | | Attention | | | | Executive Functioning | | | |
| --- | --- | --- | --- | --- | --- | --- | --- | --- | --- | --- | --- | --- |
| Comorbidity present | 0 | | 1 | | 0 | | 1 | | 0 | | 1 | |
|  | n | M (SD) | n | M (SD) | n | M (SD) | n | M (SD) | n | M (SD) | n | M (SD) |
| Hypertension | 183 | 0.06 (1.02) | 96 | -0.3 (1.02) | 181 | -0.03 (1.03) | 94 | -0.08 (1.06) | 180 | -0.03 (1.01) | 95 | -0.11 (1.1) |
| Coronary heart disease | 268 | -0.04 (1) | 11 | -0.68 (1.53) | 264 | -0.04 (1.03) | 11 | -0.2 (1.24) | 264 | -0.04 (1.04) | 11 | -0.41 (1.13) |
| Chronic heart failure | 268 | -0.05 (1.03) | 11 | -0.26 (1.14) | 264 | -0.05 (1.04) | 11 | 0 (1.06) | 264 | -0.01 (1) | 11 | -1.13 (1.52) |
| Diabetes mellitus | 265 | -0.03 (1.03) | 14 | -0.63 (1) | 261 | -0.02 (1.02) | 14 | -0.46 (1.32) | 262 | -0.02 (1.02) | 13 | -0.7 (1.3) |
| Psychiatric disorders | 239 | -0.07 (1.04 | 40 | -0.03 (0.98 | 236 | -0.01 (1.02) | 39 | -0.26 (1.14) | 236 | -0.03 (1.04) | 39 | -0.24 (1.07) |

*Note:* 0 = comorbidity not present; 1 = comorbidity present; M = mean; SD = standard deviation

Table 5: Wilcoxon Rank Sum Test with Benjamini-Hochberg False Discovery Rate correction for the domains of delayed memory, attention, and executive functioning for each comorbidity

| Domain | Comorbidity | W | p | p_adj |
| --- | --- | --- | --- | --- |
| Delayed Memory | Hypertension | 10660.5 | .003 | .045* |
| Executive Functioning | Chronic heart failure | 2126.5 | .008 | .06 |
| Delayed Memory | Diabetes mellitus | 2482 | .03 | .15 |
| Executive Functioning | Diabetes mellitus | 2259 | .042 | .158 |
| Attention | Diabetes mellitus | 2262.5 | .117 | .34 |
| Executive Functioning | Coronary heart disease | 1786 | .186 | .34 |
| Delayed Memory | Coronary heart disease | 1811 | .191 | .34 |
| Executive Functioning | Psychiatric disorders | 5178.5 | .2 | .34 |
| Attention | Psychiatric disorders | 5162.5 | .204 | .34 |
| Attention | Coronary heart disease | 1617.5 | .505 | .678 |
| Attention | Chronic heart failure | 1286.5 | .505 | .678 |
| Delayed Memory | Chronic heart failure | 1624 | .561 | .678 |
| Attention | Hypertension | 8832 | .588 | .678 |
| Executive Functioning | Hypertension | 8785.5 | .701 | .751 |
| Delayed Memory | Psychiatric disorders | 4735 | .924 | .924 |

*Note*: p_adj. = FDR-corrected p-value. * = p-value survived correction.

Table 6: Coefficient-level estimates for models fitted to estimate variation in Delayed Memory performance, taking hypertension into account

|  | |
| --- | --- |
|  | Dependent variable: |
|  |  |
|  | Delayed Memory |
|  | OLS |
|  | |
| Intercept | 9.542*** (8.296, 10.750) |
| Hospitalisation (inpatient) | **-0.702** (-1.192, -0.193)** |
| Age | **-0.024* (-0.044, -0.005)** |
| Days since infection | 0.002* (0.000, 0.003) |
| FAS | -0.018 (-0.052, 0.014) |
| PHQ-9 | -0.009 (-0.064, 0.042) |
| Hypertension | -0.133 (-0.607, 0.344) |
|  | |
| Observations | 279 |
| R2 | 0.097 |
| Adjusted R2 | 0.077 |
| Residual Std. Error | 1.701 (df = 272) |
| F Statistic | 4.882*** (df = 6; 272) |
|  | |

Note: Coefficients and 95% confidence intervals (nonparametric bootstrap, in parentheses), bolded: significant estimates, with bootstrap 95% confidence intervals not overlapping zero; *p<0.05; **p<0.01; ***p<0.001
